# Maternal Supplementation With Avocado (*Persea americana* Mill.) Pulp and Oil Alters Reflex Maturation, Physical Development, and Offspring Memory in Rats

**DOI:** 10.3389/fnins.2019.00009

**Published:** 2019-01-23

**Authors:** Marilia Ferreira Frazão Tavares de Melo, Diego Elias Pereira, Renally de Lima Moura, Elisiane Beatriz da Silva, Flávio Augusto Lyra Tavares de Melo, Celina de Castro Querino Dias, Maciel da Costa Alves Silva, Maria Elieidy Gomes de Oliveira, Vanessa Bordin Viera, Maria Manuela Estevez Pintado, Sócrates Golzio dos Santos, Juliana Késsia Barbosa Soares

**Affiliations:** ^1^Program of Food Science and Tecnology, Universidade Federal da Paraíba, João Pessoa, Brazil; ^2^Laboratory of Experimental Nutrition, Department of Nutrition, Universidade Federal de Campina Grande, Cuité, Brazil; ^3^Universidade Federal da Paraíba, João Pessoa, Brazil; ^4^Laboratory of Bromatology, Department of Nutrition, Universidade Federal da Paraíba, João Pessoa, Brazil; ^5^Laboratory of Bromatology, Department of Nutrition, Universidade Federal de Campina Grande, Cuité, Brazil; ^6^Center for Biotechnology and Chemistry, School of Biotechnology, Catholic University of Porto, Porto, Portugal

**Keywords:** avocado, cerebral fatty acids, postnatal development, memory, rats

## Abstract

Avocado (*Persea americana* Mill.) is an oleaginous fruit source of fatty acids with high levels of neuroprotective phytocomplexes. The objective of this study was to evaluate the development of reflex and somatic maturation, fatty acid profiles in the brain, and memory in different stages of life in the offspring of dams supplemented with avocado pulp and oil during gestation and lactation. The dams were randomly divided into three groups (*n* = 15 pups/group), and recieved by gavage supplementation: control group (CG)–distilled water; Avocado Oil (AO)−3,000 mg avocado oil/kg animal weight, and Avocado Pulp (AP)−3,000 mg avocado pulp/kg animal weight. We performed the following tests: Analysis of Somatic Development and Ontogeny of Postnatal Reflex (T0 to T21), the Open Field Habituation Test and the Object Recognition Test (ORT) in the adolescent (T45) and adult (T90) phases. The cerebral fatty acids content was evaluated at times T0, T21, T45, and T90. The results were analyzed using the statistical program GraphPad Prism and significant statistics were considered when *p* < 0.05. Acceleration of reflex maturation and reflex ontogeny was observed in the offspring of AO and AP fed dams, with the results being more pronounced in the pulp fed group (*p* < 0.05). All groups presented a decrease in the ambulation parameter in the second exposure to the Open Field Habituation Test, at T45 and T90 (*p* < 0.05). In the ORT, the AO and AP offspring presented memory improvements in the short and long term in the adult and adolescent phases (*p* < 0.05). The results of the brain fatty acid profiles presented higher polyunsaturated fatty acids (PUFA) content in the AO and AP groups at T21, T45, and T90. The docosahexaenoic fatty acid (DHA) content was higher at T21 (AO and AP), at T45 (AO and AP), and at T90 (AP) (*p* < 0.05). The arachidonic acid (ARA) content was higher at T45 (AO and AP), and at T90 (AO) (*p* < 0.05). Maternal supplementation with avocado oil and pulp anticipates reflex maturation and somatic postnatal development, and improves memory during the adolescent and adult phases.

## Introduction

Adequate fetal and postnatal development is influenced by maternal nutrition (Brenna and Lapillonne, 2009; Mennitti et al., 2015). During this period, considered developmentally critical, lipids are essential to tissue construction and determination of body growth (Morgane et al., 1993; Herrera and Ortega-Senovilla, 2014). Lipids structurally compose the nervous system, stimulate its development and differentiation, and even regulate neuronal cell migration (González and Visentin, 2016; Prado et al., 2018).

The quality of lipids in the diet during gestation and lactation determines the type of fatty acid (FA) that will accumulate in the fetal tissue through placental transfer and through the breast milk after birth (Lauritzen and Carlson, 2011; Innis, 2014). Fatty acids are essential nutrients for the development and maintenance of brain functions and are closely related to learning processes and memory. They demonstrate a positive correlation to neurodevelopment in the offspring through maternal lipid intake (Apryatin et al., 2017; Melo et al., 2017; Pase et al., 2017).

The principal FAs involved in brain development are polyunsaturated fatty acids (PUFAs): linoleic acid (C18: 2 ω-6) (LA), α-linolenic acid (C18: 3 ω-3) (ALA), arachidonic acid (ARA; 20:4 ω-6), docosahexaenoic acid (DHA; 22:6 ω-3), and eicosapentaenoic acid (EPA, 20:5, ω-3) (Makrides et al., 2011; González and Visentin, 2016). Since they are not endogenously synthesized, they are considered essential, and their aquisition occurs only through dietary intake of sources rich in endogenous precursors; ALA and LA (Sinclair, 1975). FA accumulating in brain tissue actively participates in the formation of neuronal membranes (Yehuda, 2012), improving learning, and memory and increasing synaptic and neurogenic plasticity (Dyall, 2017). The influence of maternal PUFAs on the development of reflexes has been evaluated in experimental studies with the offspring (Souza et al., 2012).

Non-essential FA, such as oleic monounsaturated fatty acid (18: 1 ω-9) and palmitic saturated (16: 0 ω-7), can be endogenously synthesized and also transferred through the placenta during gestation; secreted into the maternal milk and accumulate in the brain and other organs during fetal development (Innis, 2004, 2005). Oleic fatty acid is one of the main constituents of myelin (Garbay et al., 2000); it is related to axonal growth and neuronal grouping (Medina and Tabernero, 2002). Palmitic fatty acid participates in the processes of palmitoylation, gliogenesis, synaptogenesis, and myelination (González and Visentin, 2016).

Several sources of fatty acids can be used for maternal supplementation. The avocado (*Persea americana* Mill.) is an oleaginous fruit that has thus aroused scientific interest. Its lipidic composition includes monounsaturated oleic fatty acid (ω-9), saturated palmitic (ω-7), and two linoleic polyunsaturates; (ω- 6), and (ω-3) at lower levels (USDA, 2011; Dreher and Davenport, 2013). Avocado is also a source of neuroprotective antioxidant phytocomplexes (phytosterols, carotenoids, flavonoids) (Ameer, 2016).

Considering associations between maternal lipid consumption and its effects on the neurodevelopment of the offspring and the scarcity of information in the literature on the effect of avocado consumption at this stage, we hypothesized that maternal supplementation with avocado might anticipate the appearance of the reflexes and somatic maturation, and improve the offspring's memory. The objective of this research was to evaluate the offspring of dams supplemented with avocado oil and pulp during gestation and lactation for somatic and reflex development, analyze fatty acid profiles in the brain, and memory function through adulthood.

## Materials and Methods

### Avocado

Avocado (*Persea americana* Mill.) of the Hass variety was obtained from the commercial producer: Fazenda Jaguacy Avocado Brasil®, located in the municipality of Bauru, São Paulo: latitude 22°19′18″S, longitude 49°04′13″W, and 526 m altitude. Part of the fruit was used to extract oil and another part was lyophilized to obtain pulp powder. The lyophilized powder was vacuum packed, and stored at −20°C. The oil and pulp were offered by gavage starting on the seventh day of gestation and throughout the lactation period until the 21st postnatal day.

### Analysis of Fatty Acid Composition in Avocado Oil and Pulp

The fatty acid profiles of the oil and pulp were analyzed (Folch et al., 1957; Hartman and Lago, 1973) (Table 1).

**Table 1:** Fatty acid composition of avocado oil and lyophilized pulp (*Persea americana* Mill.): hass variety.

		**Avocado oil**	**Avocado pulp**
**Acids Fat**		**100 g**^**−1**^ **lipids**	
**SATURATED**			
Palmitic acid	C16:0	22.80	22.41
Stearic acid	C18:0	0.60	0.64
Araquidic acid	C20:0	0.07	0.06
Lignoceric acid	C20:4	0.07	0.08
∑ SFA		23.54	23.19
**MONOUNSATURED**			
Palmitoleic acid	C16:1ω-7	12.98	13.40
Heptadeacenoic acid	C17:1ω-7	0.10	0.09
Oleic acid	C18:1ω-9	45.92	41.66
Gondoic acid	C20:1ω-9	0.16	0.14
∑ MUFA		59.16	55.29
**POLYUNSATURED**			
Linoleic acid	C18:2ω-6	12.10	13.11
α-linolenic acid	C18:3ω-3	0.72	0.81
∑ PUFA		12.82	13.93

#### Lipidic Extraction

Sample were weighed (2 g of each) in a beaker and added to 30 ml of chloroform:methanol mixture (2:1). After this addition, the content was transferred to a deep glass container with the side covered with aluminum foil and stirred for 2 min with the help of grinder. The triturate was filtered through qualitative filter paper into a 100 ml graduated cylinder with a polished mouth. Next, the vessel walls were washed with an additional 10 mL of chloroform:methanol which was also filtered with the previous volume. The volume of the filtered extract of the graduated cylinder was recorded with the graduated cylinder closed. Twenty percentage of the final volume of the filtered extract was added to 1.5% sodium sulfate. The mixture was stirred with the graduated cylinder closed and given time for the phases to separate. It was observed that the upper phase was ~40% and the bottom 60% of the total volume. The volume of the lower phase was recorded and then the upper phase was discarded by suction with a graduated pipette. For lipid quantification, an extracted aliquot of 5 mL (lower phase) was separated with a volumetric pipette and transferred to a previously weighed beaker. This beaker was placed in an oven at 105°C so the solvent mixture could evaporate, being careful that the fat would not be degraded by heat. After cooling in a desiccator, the beaker was weighed and the fat residue weight was obtained from the difference (Folch et al., 1957).

#### Transesterification of Fatty Acids

In the sample treatment, methylation of fatty acids present in the lipid extract was carried out following the methodology described by Hartman and Lago (1973). An aliquot of the lipid extract was taken, calculated for each sample according to the fat conte2nt found in the lipid measurement, and performed according to the (Folch et al., 1957), adding 1 ml of internal standard (C19:0) and a saponification (KOH) solution. This solution was subsequently brought to heating under reflux for 4 min. Esterification solution was added immediately after, returning the solution to heating under reflux for 3 more minutes. Next, the sample was allowed to cool before subsequent washings with ether, hexane and distilled water, finally obtaining an extract (with the methyl esters and solvents), which was conditioned into a properly identified amber glass until complete drying of the solvents. After drying, a suspension in 1 ml of hexane was made and packaged into a vial for further chromatographic analysis. The aliquots of saponification and esterification solutions were determined according to the methodology described by Hartman and Lago (1973).

#### Gas Chromatography Analysis

A gas chromatograph (VARIAN 430-GC, California, EUA), coupled to a capillary column of fused silica (CP WAX 52 CB, VARIAN, California, EUA) with dimensions of 60 m × 0.25 mm and 0.25 mm film thickness was used with helium as carrier gas (Flow rate of 1 ml/min). The initial oven temperature was 100°C programmed to reach 240°C, increasing 2.5°C per minute for 30 min, totaling 86 min. The injector temperature was maintained at 250°C and the detector at 260°C. 1.0 μl aliquots of esterified extract were injected in a Split/Splitless injector. The chromatograms were recorded using Galaxie Chromatography Data System software. The fatty acids results were quantified by integration the areas of the methyl esters and are expressed in percentage by area.

### Analysis of Antioxidant Content of Oil and Lyophilized Avocado Pulp

The oil and pulp were analyzed for their total phenolic, flavonoid, and carotenoid components. The antioxidant capacity was also analyzed using the ABTS, FRAP, and IC_50_ methods.

#### Extraction

Avocado pulp constituents were extracted with both 80:20 EtOH:H_2_O v/v and evaluated for ABTS scavenging capacity, ferric reducing activity (FRAP) and total flavonoids. For total phenolic contents 100% MeOH. Oil constituents were extracted with both 80:20 MeOH:H2O v/v and evaluated for FRAP, ABTS, total phenolic and flavonoids contents. All the extractions were performed in triplicate.

#### Determination of Total Phenolic Compounds (TPC)

In order to estimate the total phenolic compounds, the methodology described by Liu et al. (2002) was used with minor modifications. The absorbance of the extract was compared with a gallic acid standard curve for estimating concentration of TPC in the sample. The TPC was expressed as mg of gallic acid equivalents (GAE) per 100 g of avocado oil and pulp on the basis of dry weight (DW).

#### Determination of Total Flavonoids

The total flavonoid content was measured using the colorimetric assay developed by Zhishen et al. (1999). The absorbance of the extract was compared with a catechin standard curve for estimating concentration of flavonoids contents in the sample. The flavonoids contents was expressed as mg of catechin equivalents (QE) per 100 g of avocado oil and pulp on the basis of dry weight (DW).

#### Antioxidant Activity–FRAP Method

The FRAP method was performed according to Benzie and Strain (1999), with modifications proposed by Pulido et al. (2000). The FRAP solution was used as reference reagent, and absorbance was read at 593 nm. The results were expressed in μmol of trolox equivalents per gram of avocado pulp on dry weight (DW) basis (μmol TE/g^−1^).

#### Antioxidant Activity–ABTS Method^+^

The ABTS method was carried out according to the methodology described by Surveswaran et al. (2007), with modifications. The results were expressed in μmol of trolox equivalent per gram of avocado oil and pulp on dry weight (DW) basis (μmol TE/g^−1^). Where A_0_ is the absorbance of the control and as is the absorbance of the sample. The effective concentration had 50% radical inhibition activity (IC_50_), expressed as mg extract/ mL, which was determined from the graph of the free radical scavenging activity (%) against the extract concentration.

The pulp and oil, respectively, presented total phenolic contents of 64.61 and 49.50 mg GAE/100 g, total flavonoids of 39.38 and 33.75 mg CE/100 g, and total carotenoids of 87.00 and 9.87 mg/100 g. For antioxidant activity, the pulp and oil presented respective FRAP values of 0.08 and 0.03 μmol TE/g, ABTS of 2.02 and 0.17 μmol TE/g, and IC_50_ of 59.86 and 443.99 mg/mL.

### Animals and Experimental Groups

Females of the Wistar lineage (90 days old/weights 250 ± 50 g) were obtained from the Laboratory of Experimental Nutrition, at the Federal University of Campina Grande–LANEX/UFCG and were breeded to obtain 45 newborn rats. The females were mated while maintained at the ratio of two females to each male. After confirmation of pregnancy, the rats were housed in individual polypropylene maternity cages (60 cm in length, 50 cm wide, and 22 cm in height), under standard laboratory conditions (temperature 22 ± 1°C, humidity 65 ± 5%, light/dark cycle of 12/12 h–artificial light from 6:00 to 18:00).

To obtain the offspring, 24 (Folch et al., 1957) female rats were randomly divided into three groups (*n* = 15 pups for each group): Control (CG)–supplemented with distilled water; Avocado Oil (AO)–supplemented with 3,000 mg of avocado oil/kg of animal weight; and Avocado Pulp (AP)–supplemented with 3,000 mg of avocado pulp/kg of animal weight. Gavage was administered from the 7th day of gestation until the 21st day of lactation: Standard feed (Presence Purina®, São Paulo, Brazil) and water was offered *ad libitum*. After weaning, the offspring received standard ration until adulthood. The research followed an experimental protocol in accordance with the ethical recommendations of the National Institute of Health (Bethesda, USA), and was approved by the ethics research committee of the Federal University of Campina Grande No: 006/2017 and avocado registered in SisGen n°A737D56.

### Experimental Procedures

**Figure 1:**
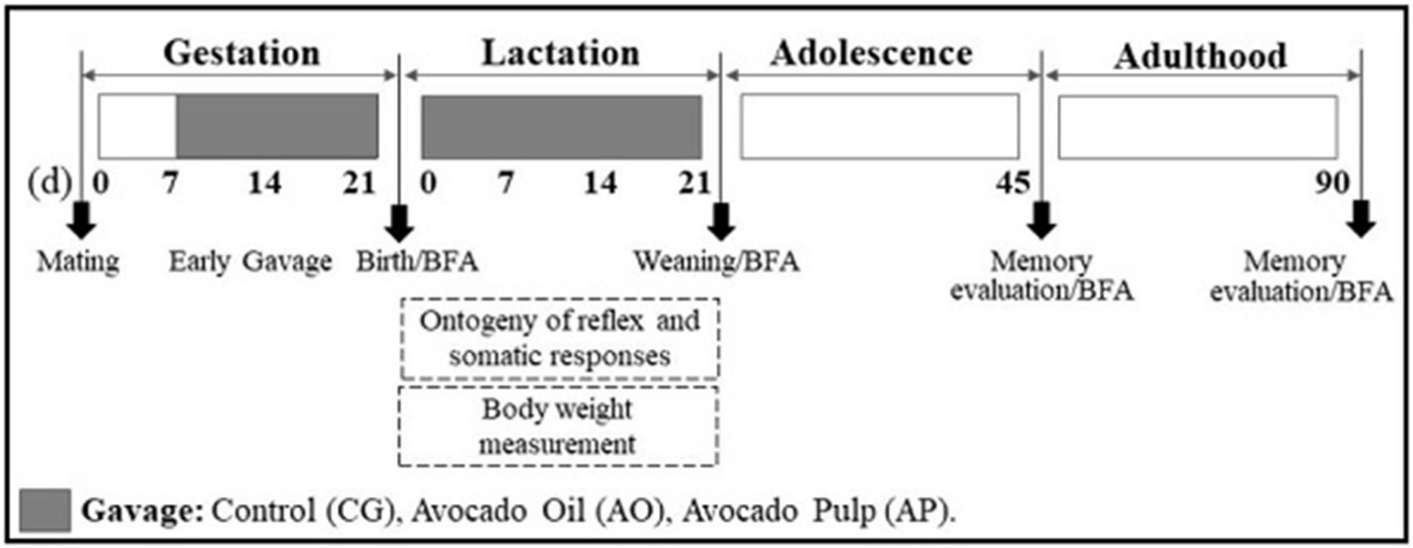
Experimental protocol. Sequence of experimental days conducted with Wistar rats supplemented during gestation and lactation, and of their offspring. (d): day; BFA: brain fatty acids.

The neonates were weighed and evaluated for reflex ontogenesis and somatic development parameters each day from birth until weaning. For fatty acid content analysis, brains were collected on the first day of life (T0), on weaning day (T21), at adolescence (T45), and as adults (T90). The memory evaluation tests were performed in adolescence and adulthood. The experimental protocol is detailed in Figure 1.

### Removal of Brains and Fatty Acid Content Analysis

At T0, after sexing and manipulation for litter reduction, surplus puppies were randomly chosen for removal of the brain, which was removed upon decapitation. At T21, T45, and T90 brains were also removed and stored at −20°C until the day of analysis and quantification of fatty acid content (*n* = 6).

The fatty acid profile of the brains was determined using the (Hartman and Lago, 1973) method with transesterification and subsequent identification by gas chromatography (Varian 430GC).

### Reflex Ontogeny and Somatic Response

Each day, from the 1st to the 21st day of life at from between 06:00 to 8:00 a.m. in the morning, somatic responses and reflex ontogeny were evaluated. The response was considered consolidated when the expected reaction was repeated for three consecutive days, being the 1st day of the appearance considered as the day of consolidation. The daily observation time for each parameter was 10 s. The reflex study followed the experimental model established by Smart and Dobbing (1971) (Table 2). Somatic maturation indicators were also evaluated: Aural “Pavilion” Opening (APO), Auditory Conduit Opening (ACO), Eye Opening (EO), Eruption of Upper Incisive Teeth (EUIT) and Inferior Teeth (EIIT), Appearance of Epidermal Hair (AEH), and Tail Length (TL).

**Table 2:** Description of the reflex test.

**Reflex**	**Stimulus**	**Response**
Palmar grasp (PG)	Light percussion on the palm of the right foreleg.	Quick bending of ankles.
Righting reflex (RR)	The rat is placed in supine position on a surface.	Return to the prone position with all paws in 10 s.
Cliff avoidance (CA)	The rat is placed on a flat and high surface (table), with legs toward the extremity.	Moves to one side and walks in the opposite direction to the edge.
Vibrissa placing (VP)	The animal is suspended by the tail and its vibrissae lightly touch the edge of a flat surface.	Both front legs are placed on the table, performing march movements.
Negative geotaxis (GN)	The rat is placed at the center of an inclined ramp with head facing downwards.	Body spin at an angle of 180°, positioning head upwards.
Auditory startle response (AS)	Intense and sudden sound stimulus.	Retraction of anterior and posterior legs, with rapid and involuntary body immobilization.
Free-fall righting (FFR)	Held by four legs at a height of 30 cm, it is released in free fall on a synthetic foam bed.	Position recovery during freefall on the surface supported by four paws.

### Memory Evaluation Tests

#### Open Field Habituation Test

During adolescent phase and adulthood the animals were submitted to the Open Field Habituation test and the Object Recognition Test (ORT). Each animal was exposed to the open field twice, in the first stage, the habituation test was performed; and after 7 (seven) days, the same test was repeated in order to compare the locomotor activity of the animals for evaluation of non-associative learning (Rachetti et al., 2012). The parameter analyzed through this test is the amount of explorative interactions taken by the animal to the field, considering the locomotion of the four legs toward the interior of each field. The test observation time was 10 min. The procedure was performed between 06:00 and 08:00 a.m., on each test day, and the sessions were filmed with a video camera. For each animal tested, the apparatus was cleaned before starting, and after completion of the test with a 10% alcohol solution.

#### Object Recognition Test (ORT)

To evaluate the short and long term memory, the Object Recognition Task (ORT) was used. The test was performed in the open field apparatus (60 × 60 × 60 cm), colored black, with six lines crossing forming 6–20 × 20 cm quadrants, uniformly lit, and with black color objects, with different shapes (rectangular or pyramid), and textures (smooth or rough) (Nava-Mesa et al., 2013).

**Figure 2:**
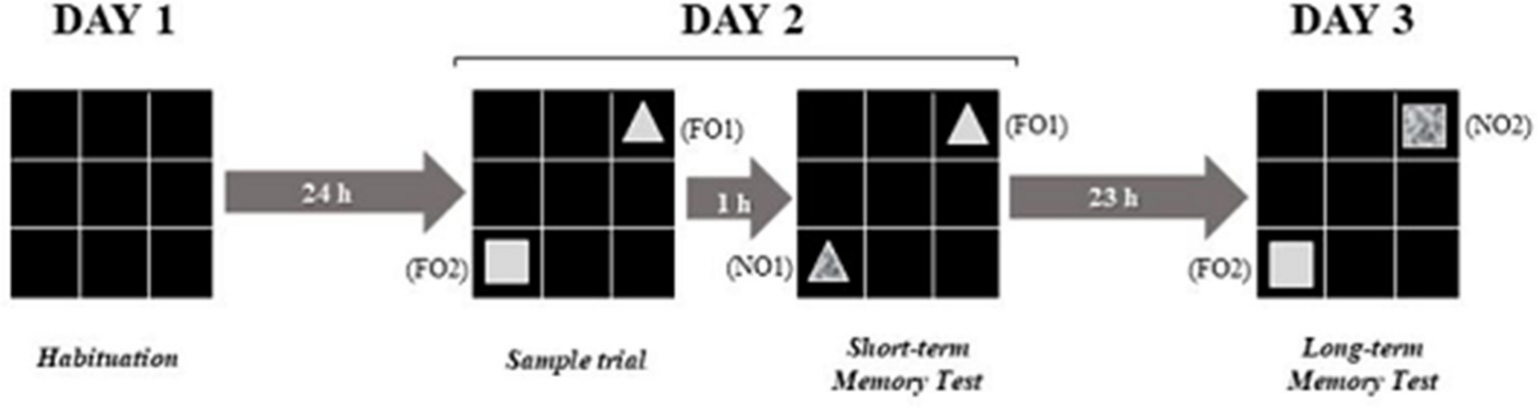
Experimental design adapted from the object recognition test. Source: adaptation of Nava-Mesa et al. (2013).

The test consisted of 4 (four) 10 min trials, taking place in 3 (three) steps: (1) Day 1–habituation for 10 min to minimize manipulation stress; (2) Day 2–performed 24 h after the habituation test, where each animal was placed in the open field containing two objects (FO1 and FO2) with identical textures (smooth), but with different forms (triangle and prismatic rectangle), located in two randomly chosen opposite corners. On the same day, yet 1 h later, the animal was placed in the open field again to explore two objects (FO1 in its original location, and a new object–NO1, identical to FO1 but with a different texture, and located in the place where FO2 had been placed during the habituation test; and (3) Day 3–was performed 24 h after the short duration test; each animal was placed in the open field to explore two objects (FO2 in its original place) and a new object (NO2) being identical to FO2 but with different texture (Figure 2).

To evaluate short-term memory, the time spent by the animal in exploring the new differently textured object (NO1) was observed. To evaluate the long-term memory, the time spent by the animal in exploring the new differently textured object (NO2) was observed at 24 h after the first exploration, on day 2. The sessions were filmed with a video camera and for each animal tested; the device was cleaned with 10% alcohol before starting and after the test. The results for the exploration times were calculated for each animal and expressed by the ratio TN/(TF + TN) TN = time spent exploring the new object; TF = time spent exploring the familiar object (Gustavsson et al., 2010; D'avila et al., 2017).

### Statistical Analysis

The results of the evaluation of reflex ontogeny and somatic development were expressed as median values for the day (Min-Max), and analyzed by Kruskal-Wallis variance analysis followed by Dunn's test (*p* < 0.05). Other results were expressed as mean ± SEM, and analyzed by ANOVA followed by Tukey (*p* < 0.05). The statistical program GraphPad Prism was used.

## Results

### Composition of Fatty Acids in Brains of Offspring After Birth

The composition of saturated fatty acids in the AO group offspring brains on the first day of life presented reduced myristic, palmitic, and stearic fatty acids as compared to the CG and AP groups (*p* < 0.05); the AP group offspring presented lower levels of myristic and higher levels of palmitic fatty acids as compared to CG offspring (*p* < 0.05) (Table 3).

**Table 3:** Composition of fatty acids present in the brain puppies (T0 and T21) of dams supplemented with oil and avocado pulp.

	**Groups**					
	**Brain**–**T0 day of life**			**Brain**–**T21 day of life**		
**Fatty acids**	**CG**	**AO**	**AP**	**CG**	**AO**	**AP**
**SATURATED**						
Myristic acid **C14:0**	1.51 ± 0.05	1.15 ± 0.04^*^	1.40 ± 0.05^*^^#^	0.40 ± 0.00	0.32 ± 0.01^*^	0.43 ± 0.11^*^^#^
Palmitic acid **C16:0**	25.27 ± 0.30	22.57 ± 0.20^*^	26.16 ± 0.40^*^^#^	20.11 ± 0.05	17.58 ± 0.07^*^	18.88 ± 1.01^*^^#^
Stearic acid **C18:0**	15.63 ± 0.10	13.40 ± 0.04^*^	15.01 ± 0.09^*^^#^	17.90 ± 0.50	15.16 ± 0.64^*^	15.92 ± 0.84^*^^#^
Behenic acid **C22:0**	–	–	–	0.16 ± 0.01	0.13 ± 0.02^*^	0.12 ± 0.01^*^
Total	42.41	37.12^*^	42.57^*^	38.57	33.19^*^	35.35^*^^#^
**MONOUNSATURATED**						
Palmitoleic acid **C16:1ω7c**	3.74 ± 0.03	1.48 ± 0.02^*^	3.93 ± 0.03^*^^#^	0.69 ± 0.00	0.47 ± 0.01^*^	0.39 ± 0.02^*^^#^
Vaccenic acid **C18:1ω7c**	2.90 ± 0.02	2.63 ± 0.03^*^	3.03 ± 0.01^*^^#^	2.91 ± 0.10	2.54 ± 0.18^*^	2.76 ± 0.22^#^
Oleic acid **C18:1ω9**	11.12 ± 0.10	9.86 ± 0.09^*^	11.41 ± 0.10^*^^#^	12.96 ± 0.20	10.83 ± 0.26^*^	11.74 ± 1.19^*^^#^
Gondoic acid **C20:1ω9**	0.20 ± 0.02	0.22 ± 0.01^*^	0.20 ± 0.02^#^	0.52 ± 0.04	0.60 ± 0.06	0.67 ± 0.12^*^
Erucid acid **C22:1ω9**	–	–	–	0.09 ± 0.01	0.06 ± 0.01^*^	0.07 ± 0.03
Total	17.96	14.19^*^	18.57^*^^#^	17.17	14.50^*^	15.56^*^^#^
**POLYUNSATURATED**						
Linoleic acid **C18:2ω6c**	1.04 ± 0.10	0.78 ± 0.07^*^	0.86 ± 0.09^*^^#^	1.37 ± 0.10	0.93 ± 0.08^*^	0.94 ± 0.16^*^
Eicosadienoic acid **C20:2ω6**	1.01 ± 0.10	0.15 ± 0.01^*^	0.16 ± 0.01^*^^#^	0.27 ± 0.02	0.20 ± 0.02^*^	0.20 ± 0.04^*^
Dihomo-γ-linolenic acid **C20:3** **ω6**	0.46 ± 0.20	0.58 ± 0.30^*^	0.61 ± 0.40^*^^#^	0.39 ± 0.01	0.39 ± 0.04	0.39 ± 0.07
Arachidonic acid **C20:4ω6c**	10.15 ± 0.10	8.67 ± 0.09^*^	10.14 ± 0.12^#^	10.18 ± 0.20	8.76 ± 0.35^*^	9.27 ± 0.44^*^^#^
Docosatetraenoic acid **C22:4** **ω6**	3.27 ± 0.02	2.60 ± 0.28^*^	2.64 ± 0.20^*^^#^	3.48 ± 0.02	2.47 ± 0.09^*^	3.11 ± 0.56^#^
Docosapentaenoic acid **C22:5** **ω3**	2.71 ± 0.09	4.00 ± 0.12^*^	3.35 ± 0.10^*^^#^	0.78 ± 0.10	4.17 ± 0.02^*^	6.99 ± 0.04^*^^#^
Docosahexaenoic acid **C22:6ω3**	8.19 ± 0.32	6.35 ± 0.23^*^	6.78 ± 0.40^*^^#^	10.74 ± 0.00	12.17 ± 1.69^*^	13.98 ± 0.9^*^^#^
Total	26.83	23.13^*^	24.27^*^^#^	27.31	31.00^*^	34.88^*^^#^
**SUMS AND RATIONS**						
PUFA/SFA	0.63	0.62^*^	0.57^*^^#^	0.47	0.93^*^	0.99^*^^#^
**ω3**	11.36	10.93^*^	10.47^*^^#^	12.01	18.64^*^	21.36^*^^#^
**ω6**	15.47	12.20^*^	13.80^*^^#^	15.30	12.36^*^	13.52^*^^#^
**ω9**	0.20	0.22^*^	0.20^#^	0.61	0.66^*^	0.67^*^
**ω6/ω3**	1.36	1.12^*^	1.32^*^	1.27	0.67^*^	0.63^*^^#^

*Data expressed as mean ± standard deviation. CG, Control Group; AO, Avocado Oil Group; AP, Avocado Pulp Group*.

* *vs. CG*.

# *vs. AO*.

*T0, at birth; T21, at weaning (21 days of life). Statistical test used was One way Anova, followed by Tukey with a (p < 0.05) level of significance*.

Palmitoleic, vaccenic e oleic (monounsaturates) were also found decreased in the AO group offspring as compared to the CG and AP group offspring (*p* < 0.05). However, the AP groups presented higher values for these fatty acids then the CG and AO (*p* < 0.05) (Table 3).

The total PUFA content was 15% lower in the AO group offspring (10% lower in the AP group) as compared to the CG offspring. The AO offspring presented reductions in linoleic, eicosadienoic, arachidonic, docosatetraenoic, and docosahexaenoic polyunsaturated fatty acids as compared to the CG and AP offspring (*p* < 0.05). AP offspring also presented reductions in linoleic, eicosadienoico, docosatetraenoic, and docosahexaenoic acids as compared to the CG offspring (*p* < 0.05). However, eicosatrienoic and docosapentaenoic acid levels were higher in the AP and AO offspring brains compared to the CG offspring (*p* < 0.05) (Table 3).

### Composition of Fatty Acids in Offspring Brains at the end of Lactation (21 Days of Life)

At 21 days of age, myristic, palmitic, stearic, and behenic saturated fatty acids levels were found decreased in the AO and AP group offspring brains when compared to the CG (*p* < 0.05). The AP group presented higher levels of these fatty acids than the AO group (*p* < 0.05) (Table 3).

Both AO and AP groups presented lower total MUFA values, with reductions in palmitoleic, vaccenic, oleic, and erucic fatty acids in AO brains compared to the CG. For palmitoleic and oleic fatty acids, the AP group brains also presented lower total values as compared to the CG and (*p* < 0.05). Gondoic acid alone was higher in the AP group as compared to the CG (*p* < 0.05) (Table 3).

The polyunsaturates (linoleic, eicosadienoic, arachidonic, and docosatetraenoic acid) were decreased in the AO and AP brains as compared to the CG (*p* < 0.05). However, total PUFAs were, respectively, 13.5 and 28% higher in the AO and AP groups as compared to the CG; due to the increased DHA and docosapentaenoic acid levels. Also the total PUFAs were higher in the AP group when compared to the AO group (*p* < 0.05) (Table 3).

### Composition of Fatty Acids in Offsprings' Brains in Adolescence (45 Days of Life)

Saturated fatty acid levels in the offspring brains (adolescents) were similar for all groups; except for behenic acid, which was higher in the AO and AP groups as when compared to the CG (*p* < 0.05) (Table 4).

**Table 4:** Composition of fatty acids present in the brain offspring (T45 and T90) of dams supplemented with oil and avocado pulp.

	**Groups**					
	**Brain**–**T45 day of life**			**Brain**–**T90 day of life**		
**Fatty acids**	**CG**	**AO**	**AP**	**CG**	**AO**	**AP**
**SATURATED**						
Myristic acid **C14:0**	0.12 ± 0.04	0.11 ± 0.00	0.12 ± 0.01	0.13 ± 0.02	0.13 ± 0.02	0.11 ± 0.01
Palmitic acid **C16:0**	15.82 ± 2.60	14.89 ± 1.14	16.22 ± 2.11	16.11 ± 0.90	17.83 ± 0.42^*^	16.28 ± 1.73^#^
Stearic acid **C18:0**	16.21 ± 2.91	15.29 ± 1.02	15.89 ± 1.11	16.42 ± 0.94	18.65 ± 0.21^*^	17.04 ± 1.62^#^
Behenic acid **C22:0**	0.19 ± 0.03	0.24 ± 0.02^*^	0.22 ± 0.01^*^	0.25 ± 0.02	0.33 ± 0.01^*^	0.27 ± 0.05^#^
Lignoceric acid **C24:0**	–	0.15 ± 0.01	0.16 ± 0.01^#^	0.19 ± 0.01	0.19 ± 0.01	0.19 ± 0.01
Total SAT	32.34	30.68^*^	32.61^*^^#^	33.10	37.13^*^	33.89^*^^#^
**MONOUNSATURATED**						
Palmitoleic acid **C16:1ω7c**	0.34 ± 0.02	0.30 ± 0.05	0.45 ± 0.29	0.30 ± 0.03	0.27 ± 0.04	0.25 ± 0.07
Vaccenic acid **C18:1ω7c**	3.22 ± 0.61	3.09 ± 0.07	3.32 ± 0.34	3.65 ± 0.34	4.07 ± 0.06	3.78 ± 0.66
Oleic acid **C18:1ω9**	13.37 ± 2.28	13.05 ± 0.19	13.95 ± 1.39	15.47 ± 1.36	17.24 ± 1.21	16.23 ± 2.52
Gondoic acid **C20:1ω9**	1.66 ± 0.28	1.72 ± 0.16	1.79 ± 0.02	2.72 ± 0.35	3.16 ± 0.11	2.99 ± 0.91
Erucid acid **C22:1ω9**	0.17 ± 0.08	0.20 ± 0.06	0.17 ± 0.07	0.29 ± 0.00	0.38 ± 0.03^*^	0.27 ± 0.11^#^
Total Monounsat	18.69	18.36^*^	19.68^*^^#^	22.43	25.11^*^	23.52^*^^#^
**POLYUNSATURATED**						
Linoleic acid **C18:2ω6c**	0.59 ± 0.13	0.63 ± 0.08	0.61 ± 0.11	0.61 ± 0.00	0.67 ± 0.04^*^	0.53 ± 0.06^*^^#^
Eicosadienoic acid **C20:2ω6**	0.23 ± 0.06	0.22 ± 0.00	0.22 ± 0.02	0.22 ± 0.01	0.25 ± 0.00^*^	0.12 ± 0.02^*^^#^
Dihomo-γ-linolenic acid **C20:3** **ω6**	0.35 ± 0.07	0.36 ± 0.03	0.39 ± 0.00	0.28 ± 0.02	0.36 ± 0.01^*^	0.24 ± 0.02^#^
Arachidonic acid **C20:4ω6c**	6.41 ± 0.43	7.09 ± 0.52	8.06 ± 0.64^*^^#^	7.23 ± 0.61	8.17 ± 0.74^*^	7.59 ± 0, 10
Docosatetraenoic acid **C22:4** **ω6**	3.20 ± 0.70	3.17 ± 1.02	3.90 ± 0.59	2.74 ± 0.23	2.79 ± 0.16	2.80 ± 0.18
Docosapentaenoic acid **C22:5** **ω3**	3.28 ± 0, 69	4.60 ± 0.89^*^	3.28 ± 0.85^#^	1.43 ± 0.13	1.40 ± 0.02	1.40 ± 0.09
Docosahexaenoic acid **C22:6ω3**	11.39 ± 1.37	15.86 ± 1.03^*^	16.76 ± 1.52^*^	11.45 ± 1.24	10.48 ± 0.43	15.80 ± 0.97^*^^#^
Total	25.45	31.93^*^	33.22^*^^#^	23.96	24.12^*^	28.48^*^^#^
**SUMS AND RATIONS**						
PUFA/SFA	0.79	1.04^*^	1.02^*^^#^	0.72	0.65^*^	0.84^*^^#^
**ω3**	15.02	20.82^*^	20.43^*^^#^	13.16	12.24^*^	17.44^*^^#^
**ω6**	10.43	11.11^*^	12.79^*^^#^	10.80	11.88^*^	11.04^*^^#^
**ω9**	1.83	1.92^*^	1.96^*^^#^	3.01	3.54^*^	3.26^*^^#^
**ω6/ω3**	0.69	0.53^*^	0.63^*^^#^	0.82	0.97^*^	0.63^*^^#^

*Data expressed as mean ± standard deviation. CG, Control Group; AO, Avocado oil Group; AP, Avocado Pulp Group of*.

* *vs. CG*.

# *vs. AO*.

*T45, adolescent phase (45 days of life); T90, adult phase (90 days of life). Statistical test used was One Anova way followed by Tukey with a level of significance of (p < 0.05)*.

There was no difference for monounsaturated acid contents. However, total PUFAs were 48.85% higher in the AO brains and 54.77% in the AP brains than in the CG brains. Compared to the CG brains, increased levels of DHA and docosapentaenoic fatty acid were found in the AO brains; and arachidonic, and docosahexaenoic acids were higher in the PA brains (*p* < 0.05) (Table 4).

### Composition of Fatty Acids in the Adult Offspring Brain (90 Days of Life)

In adulthood, the content of saturated palmitic, stearic and behenic fatty acids in the AO offspring group brains was higher than the AP or control groups (*p* < 0.05). In relation to monounsaturated fatty acids, vacênic acid was different between the groups, with higher levels in the AO and AP brains as compared to the CG (*p* < 0.05). Monounsaturate erucic acid was higher in the AO brain as compared to the AP group and the controls (*p* < 0.05) (Table 4).

Linoleic, eicosadienoic and eicosatrienoic polyunsaturates presented higher levels in the AP offspring than in the AP and CG offspring (*p* < 0.05). Arachidonic acid was higher in the AO groups as compared to the CG, and docosahexaenoic acid presented higher levels in the AP group as compared to the AO and control groups (*p* < 0.05) (Table 5). Total PUFAs were higher in the brains of the AP (22%) groups as compared to the controls (Table 4).

**Table 5:** Reflex maturation in offspring of mothers supplemented with avocado oil and pulp during gestation and lactation.

		**Groups**	
**Reflexes**	**CG**	**AO**	**AP**
Palmar grasp (PG)^a^	8 (6–13)	5 (3–7)^*^	4 (3–5)^*^
Righting reflex (RR)	4 (1–9)	4 (2–7)	4 (2–6)
Vibrissa placing (VP)^b^	10 (5–13)	9 (7–10)	7 (7–10)^*^^#^
Cliff avoidance (CA)^b^	10 (6–15)	6 (5–8)^*^	6 (2–10)^*^
Negative geotaxis (GN)^b^	20 (19–21)	13 (12–14)^*^	10 (10–12)^*^^#^
Auditory tartle response (AS)^b^	12 (11–13)	13 (12–13)	11 (10–12)^*^^#^
Free-fall righting (FFR)^b^	12 (8–15)	7 (2–14)^*^	5 (2–9)^*^

*Data were expressed as mean values of the day (Min-Max) and analyzed by Kruskal-Wallis analysis of variance followed by Dunn's test (p < 0.05)*.

* *Compared to control group*.

# *Compared to the avocado oil group*.

Considering:

a Day of response disappearance and

b *Day of response appearance*.

*CG (Control Group-n = 15), AO (Avocado Oil Group-n = 15), AP (Avocado Pulp Group-n = 15)*.

### Body Weight and Tail Length

**Figure 3:**
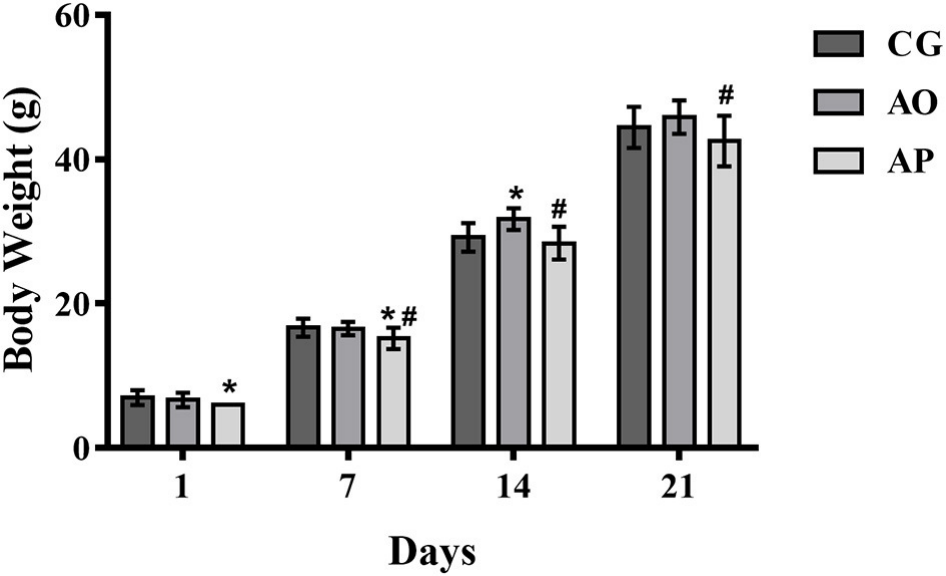
Mean body weight in grams (g) (±SEM) of neonatal rats whose mothers received supplementation with avocado oil and pulp (3,000 mg/kg body weight) during gestation and lactation. ANOVA followed by Tukey (*p* < 0.05). (*) statistically different as compared to CG; (#) statistically different as compared to AO. CG (Control Group-*n* = 15), AO (Avocado Oil Group-*n* = 15), AP (Avocado Pulp Group-*n* = 15).

The body weight results for offspring of mothers treated with avocado oil and pulp during gestation and lactation are shown in Figure 3. The weights of the offspring of the pulp group (AP) were significantly lower than the control group (CG) during the first week of lactation (1st and 7th day), and when compared to the oil group (AO), the weights were lower from the 7th to the 21st day (*p* < 0.05). Only on the 14th day of lactation did the AO pups present significantly higher weights as compared to the CG (*p* < 0.05). By the end of lactation, the differences differences did not persist.

**Figure 4:**
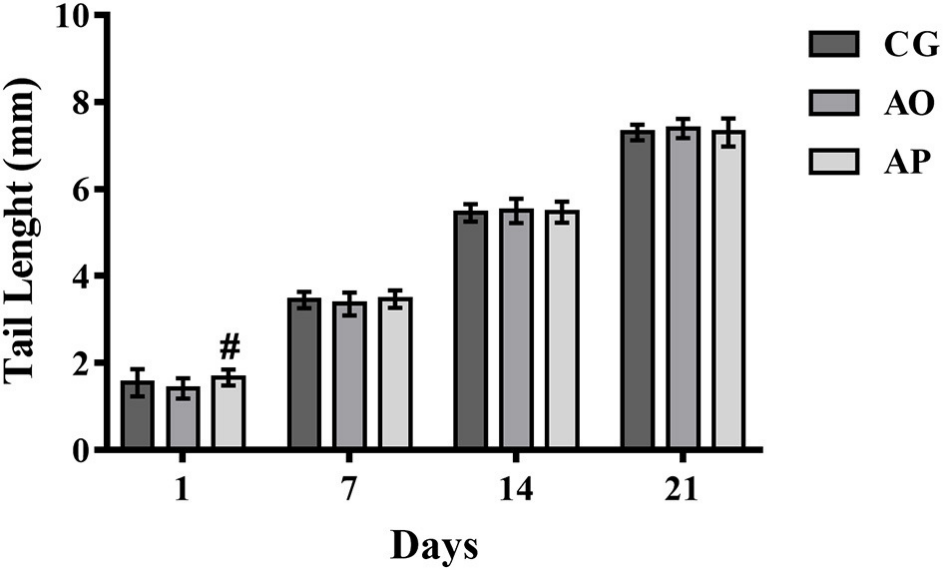
Tail lengths of the offspring of mothers supplemented with avocado oil and pulp (3,000 mg/kg body weight) during gestation and lactation. Data expressed as mean ± SEM and analyzed by ANOVA followed by Tukey (*p* < 0.05). (#) statistically different as compared to the AO group. CG (Control Group-*n* = 15), AO (Avocado Oil Group-*n* = 15), AP (Avocado Pulp Group-*n* = 15).

The tail lengths presented significant differences only on the first day of life, where the AP pupils presented larger sizes as compared to the AO group (*p* < 0.05) (Figure 4). The difference did not remain beyond the 7th day (through the end of lactation).

### Ontogenesis of Reflex, and Somatic Maturation

The offspring of mothers supplemented with pulp (AP) compared to the CG presented early disappearance of the PG, and appearance of the following reflexes: VP, CA, GN, AS, and FFR (*p* < 0.05). These same pups also anticipated the VP, GN, and AS reflexes as compared to the AO group (*p* < 0.05). The AO offspring, in relation to the CG, presented early PG onset, and the appearance of CA, GN, and FFR (*p* < 0.05) (Table 5).

For the somatic indicators, the AP neonates presented anticipation in auditory conduction opening and epidermic hair appearance, yet delayed eruption of inferior incisors as compared to the CG (*p* < 0.05). The same group (AP) when compared to the AO group presented anticipated auditory conduit opening together with superior incisor eruption (*p* < 0.05). The neonates of the AO group presented auditory conduction opening and inferior incisor eruption delays when compared to the CG (*p* < 0.05) (Table 6).

**Table 6:** Somatic development in offspring of mothers supplemented with avocado oil and pulp during gestation and lactation.

		**Groups**	
**Physical characteristics**	**CG**	**AO**	**AP**
Ear unfolding	3 (2–4)	3 (2–4)	3 (2–4)
Auditory conduit opening	14 (13–15)	13 (12–13)^*^	11 (10–12)^*^^#^
Eye opening	14 (12–15)	13 (12–15)	14 (12–15)
Eruption of superior incisors	10 (8–12)	11 (9–12)	9 (8–11)^#^
Eruption of inferior incisors	4 (2–5)	8 (8–9)^*^	7 (7–8)^*^
Epidermic hair appearance	3 (2–4)	3 (3–3)	3 (2–3)^*^

*Data were expressed as mean values of the day (Min-Max), analyzed by Kruskal-Wallis analysis of variance, followed by Dunn's test (p < 0.05)*.

* *Compared to the control group*.

# *Compared to the avocado oil group*.

*CG (Control Group-n = 15), AO (Avocado Oil Group-n = 15), AP (Avocado Pulp Group-n = 15)*.

### Behavioral Testing

#### Open Field

**Figure 5:**
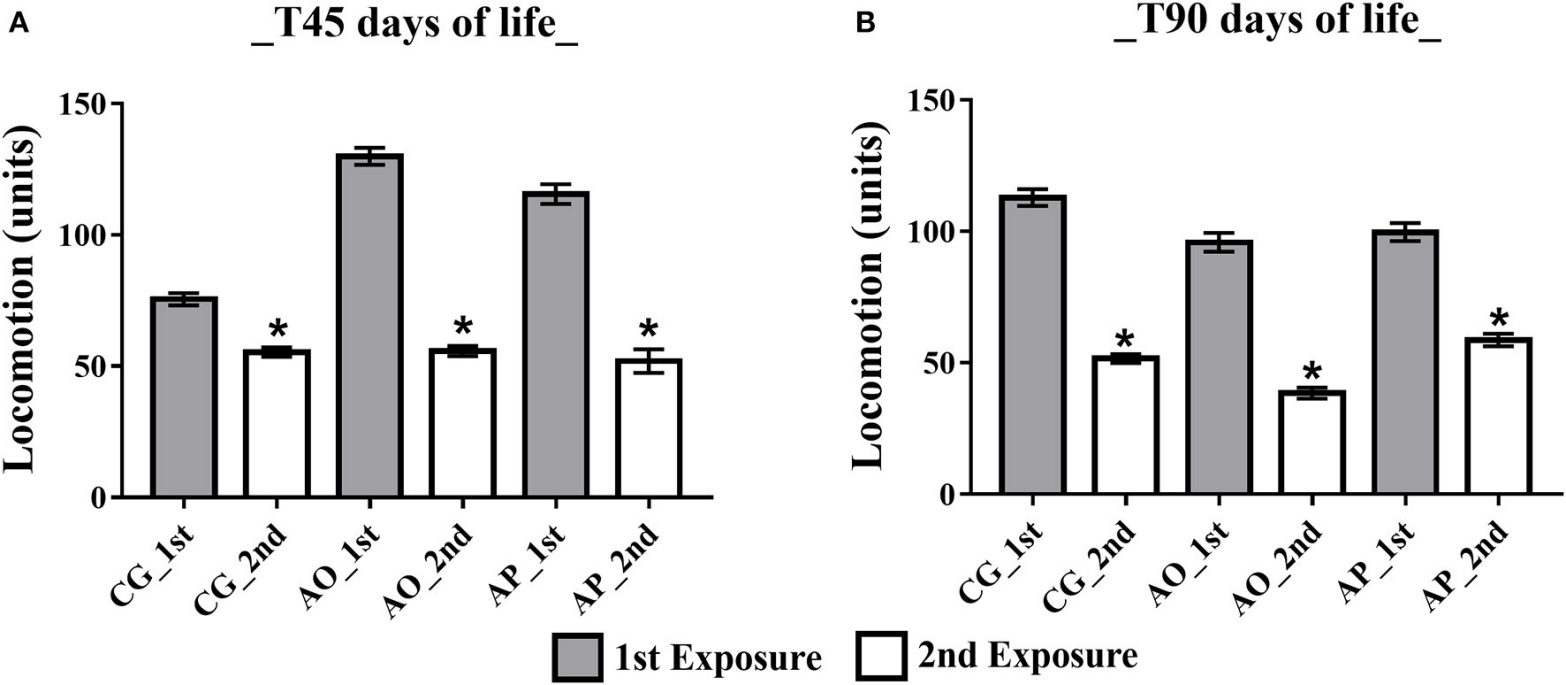
Influence of maternal supplementation with avocado oil and pulp on total offspring ambulation. Data expressed as mean and standard error (±SEM), analyzed by ANOVA, and followed by Tukey (*p* < 0.05). **(A)** Adolescent phase offspring (T45); **(B)** Adult offspring (T90). 1st: first exposure; 2nd: second exposure. CG (Control Group-*n* = 15), AO (Avocado Oil Group-*n* = 15), AP (Avocado Pulp Group-*n* = 15). *p < 0.05 vs. 1st exposure in the open field.

Open Field Habituation Test ambulatory analysis at 45 days (adolescent stage) presented differences between the first and second exposures, with a decrease in the ambulation parameter during the second exposure for all groups: CG (77. 50 ± 5.75 and 55.36 ± 5.44), AO (129.92 ± 11.16 and 55.75 ± 5.44), and AP (115.56 ± 11.13 and 56.25 ± 5.10) (*p* < 0.05) (Figure 5A). In the adult phase (T90) the same differences persisted, yet with ambulation exposure decreases in the CG (112.82 ± 10.57 and 51.67 ± 5.57), AO (95.83 ± 8.77 and 38.50 ± 4.97), and AP (99.71 ± 9.09 and 60.50 ± 5.95) (*p* < 0.05) (Figure 5B).

#### Object Recognition Test (ORT)

##### Adolescent phase

**Figure 6:**
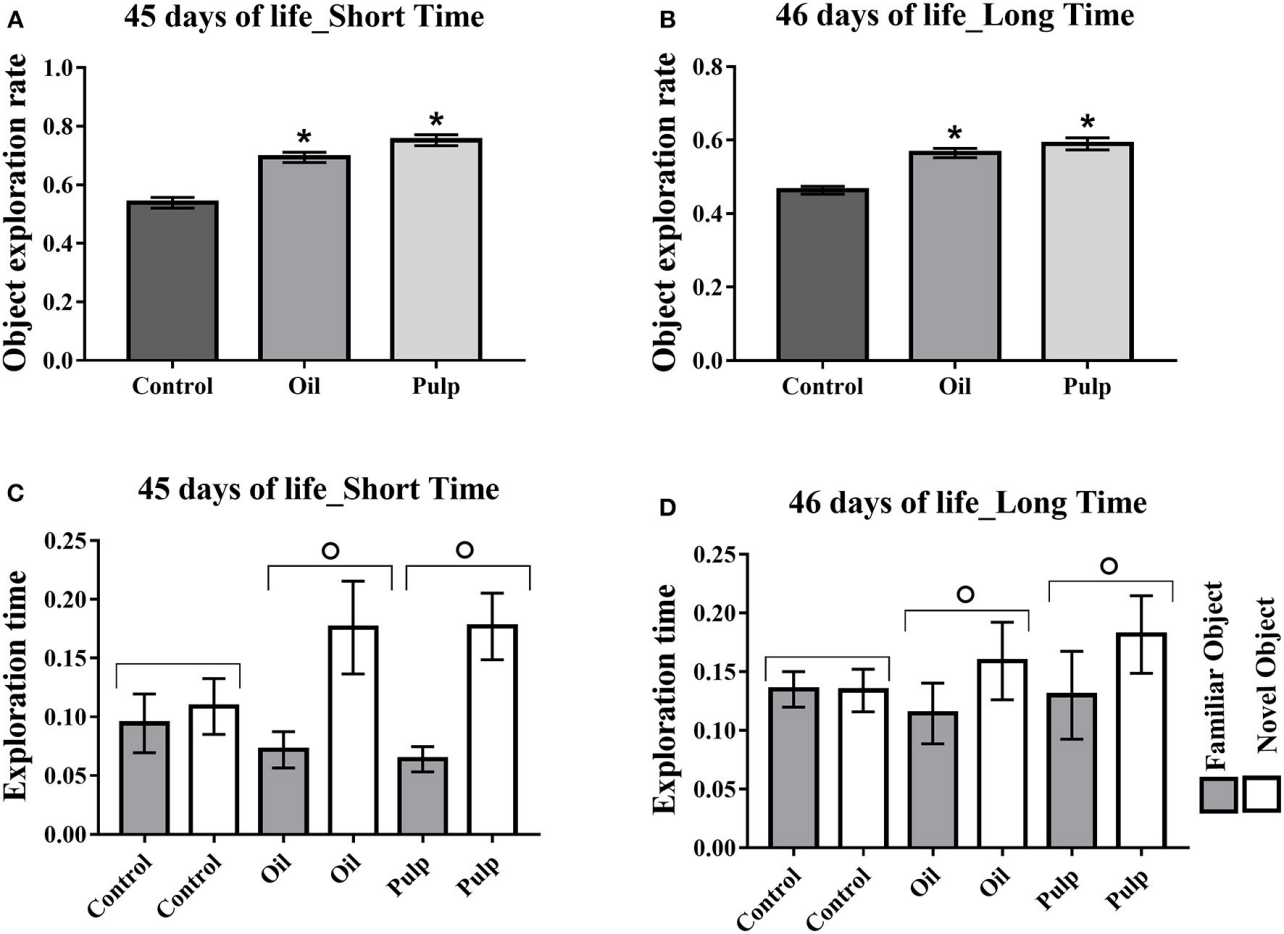
Adolescent offspring; short and long term memory test; preference in the object exploration. Data expressed as mean and standard error (±SEM) **(A,B)** and mean and standard deviation (±SD) **(C,D)** Analyzed by ANOVA, and followed by Tukey (*p* < 0.05). **(A)** Object exploration rate short term. **(B)** Object exploration rate long term. **(C)** Time of exploration of the family object and new object in the short term. **(D)** Time of exploration of the familiar object and new object in the long term. *Indicates a significant difference between the AO and AP groups vs. the CG in new object exploration time (*p* < 0.05). **°**Indicates a significant difference for the same group, in the time of exploration of the familiar object and the new object. Control (Control Group-*n* = 15), Oil (Avocado Oil Group-*n* = 15), Pulp (Avocado Pulp Group-*n* = 15).

In the adolescent phase, the rate of new object exploration in the short term and in the long term tests was higher in the AO and AP groups, presenting higher exploration rates as compared to the CG (*p* < 0.05) (Figures 6A,B). The groups AO and AP presented greater time for the new object, relative to the familiar object, in both short and long periods (*p* < 0.05) (Figures 6C,D).

##### Adulthood

**Figure 7:**
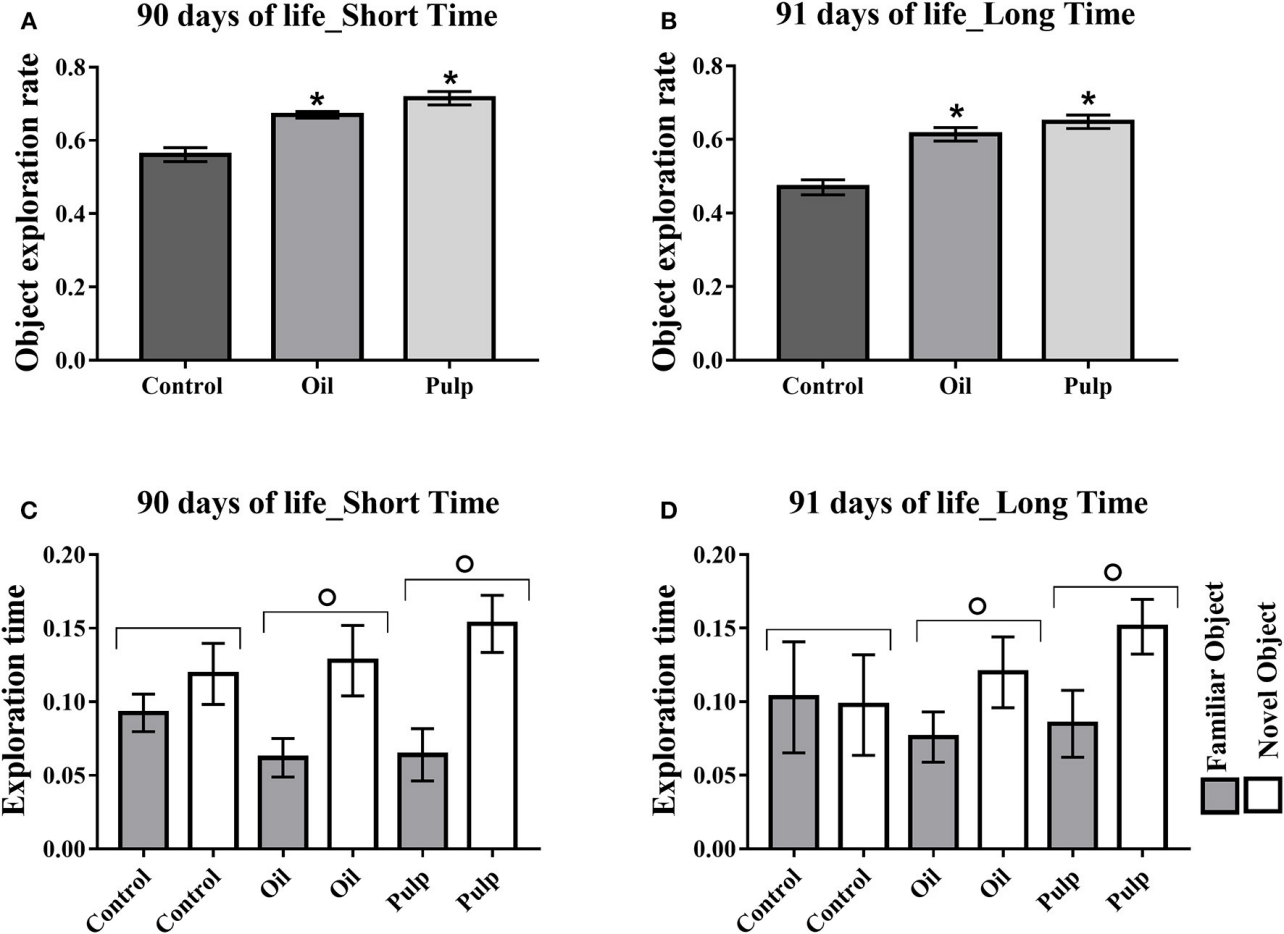
Exploration of test objects (short and long-term memory) in adult offspring. Data expressed as mean and standard error of the mean (±SEM), **(A,B)** and mean and standard deviation (±SD) **(C,D)** analyzed by ANOVA, followed by Tukey (*p* < 0.05). **(A)** Short term memory object exploration test. **(B)** Long-term memory object exploration test. **(C)** Time of exploration of the family object and new object in the short term. **(D)** Time of exploration of the familiar object and new object in the long term. *Indicates a significant difference between the AP and AO groups vs. the CG in new object exploration time. **°**Indicates a significant difference for the same group, in the time of exploration of the familiar object and the new object. Control (Control Group-*n* = 15), Oil (Avocado Oil Group-*n* = 15), Pulp (Avocado Pulp Group-*n* = 15).

Adult offspring in the AP and AO groups also presented higher rates of new object exploration in the short and long term (*p* < 0.05) (Figures 7A,B). The groups AO and AP presented greater time for the new object, relative to the familiar object, in both short and long periods (*p* < 0.05) (Figures 7C,D).

## Discussion

Maternal fatty acids transferred via the placenta, and through the breast milk are considered critical for growth and development (Lauritzen and Carlson, 2011; Innis, 2014). Thus, during gestation and lactation, manipulation of lipids can affect the availability of fatty acids to the fetus and the infant. In the present study, avocado oil and pulp supplementation during gestation and lactation positively influenced the offspring in: (1) reflex development, (2) somatic maturation, (3) and memory acquisition (4) the fatty acid profiles of the brains of the neonates, adolescents, and adult offspring.

Maternal consumption of distinct lipids presents differing consequences for the weight, growth, and somatic parameters in their offspring. At birth, and during the first week of lactation, the results reveal that the offspring of the AP mothers had lower weights than the CG. Several studies have reported a decrease in the body weights of offspring with mothers receiving lipids from differing sources; at times presenting similar lipid profiles as compared to those used in the present research, such as cashew nuts (Melo et al., 2017), which has fiber as Avocado pulp and Buriti oil (*Mauritia flexuosa*) (Medeiros et al., 2015), and olive oil (Sánchez et al., 2012; Priego et al., 2013), which are source of polyunsaturated fatty acids as the lipids used in the present research (pulp and avocado oil). The presence of fiber and polyunsaturated fatty acids in maternal diet can induce reduction of plasma triglycerides (TG). Increased maternal levels have been used as a biochemical marker to increase offspring birth weight (Barbour and Hernandez, 2018) and their lower plasma levels have been associated with improved insulin sensitivity and lower caloric influx (Nolan et al., 1995). In contrast, diets with high levels of SFA induce an increase in plasma triglycerides, consequently, they also can induce increase in the offspring weight (Ferro Cavalcante et al., 2013; Soares et al., 2013; Cadena-Burbano et al., 2017). Therefore, it was observed in the present research a reduction in TG at the end of the lactation of the mothers fed with the pulp, when compared to the others groups (data not shown). These findings are in agreement with Barbour and Hernandez (2018).

Maternal supplementation with avocado promoted acceleration in the postnatal appearance of several somatic parameters. Lipids are recognized for promoting somatic growth in the offspring (Del Prado et al., 1997). Both avocado oil and pulp, despite having high amounts of oleic and palmitoleic acids in their composition, have ω-6 and ω-3 fatty acids, which have been associated with physical growth in rat progeny (Santillán et al., 2010; Ferro Cavalcante et al., 2013). These results are consistent with experiments that used PUFA and MUFA in the source foods (Ferro Cavalcante et al., 2013; Melo et al., 2017), and the same for avocado lipids, yet results diverge for SFA-source diets (Soares et al., 2009). The findings confirm that both the quality and amount of lipids in the maternal diet directly influence physical development in the offspring (Hausman et al., 1991). DHA and ARA (in combination) are essential for optimal growth and development early in life (Harauma et al., 2017).

During the critical developmental phase, essential fatty acids are needed for physical growth and good brain development. The brain goes through processes that include neural network organization; accumulation of DHA and ARA occurs to support active neurogenesis and neuronal growth (Lauritzen and Carlson, 2011; Innis, 2014), while modifying the fluidity and signaling of neuronal membranes (Bazinet and Layé, 2014). In this period, specific brain regions, including the hippocampus, striatum, visual and auditory cortices respond similarly to nutritional insults (Kretchmer et al., 1996), leading to long-term effects (Morgane et al., 1993; Arcego et al., 2017).

Our results showed that at the end of gestation, or at the beginning of the postnatal phase (T0), there was less incorporation of DHA (C22: 6n3) in the brains of the AO and AP offspring. However, by the end of lactation, levels of DHA had increased in brains of the AO and AP offspring as compared to the control groups. In rodents, fetal demand for fatty acid incorporation occurs from the last week of gestation to the end of lactation (Morgane et al., 2002). This explains the observed increase in DHA incorporation in the T21 brain levels, as compared to T0 levels. Accumulation of fatty acids in the offsprings' brains is influenced by pre-fetal and post-fetal maternal supply (Innis, 2011). Avocado oil and pulp present low linolenic acid content (ALA); a DHA precursor. The increases observed in the brain levels for this fatty acid in the offspring of mothers who consumed avocado oil and pulp oppose studies that have found a positive relation between low ALA content and low proportions of DHA in offspring brain tissue (Amusquivar et al., 2000; Melo et al., 2017; Lopez-Soldado et al., 2018). However, avocado presents high phospholipid (PL) content, present in the lipid fraction of its pulp (Cowan and Wolstenholme, 2016; Pacetti et al., 2017) and oil (Takenaga et al., 2008). Increases in DHA uptake in the brains of the AO and AP offspring can be explained by the presence of phospholipids in avocado. DHA is synthesized by ALA desaturation and stretching reactions (Pereira et al., 2003; Novak et al., 2008), and when esterified into PLs, is more efficiently incorporated into brain tissue (Murru et al., 2013; Kitson et al., 2016; Destaillats et al., 2018). Of the phospholipids, lyso-phosphatidylcholine (LPC) as esterified to DHA (LPC-DHA) is the most efficient way to cross the blood-brain barrier inducing a greater deposition of DHA in the brain (Nguyen et al., 2014). In the fetal brain formation and postnatal development periods, LPC-DHA is associated with an increase in exogenous PUFA uptake and deposition in the membranes of brain tissue, which promotes higher DHA deposition (Chan et al., 2018). One study reveals that offspring of mothers fed LPC from DHA-enriched eggs present higher levels of this FA in certain brain regions (Valenzuela et al., 2010). These findings are similar to the data found in the present study.

Reflex ontogeny is another parameter used to evaluate development because it measures maturation and central nervous system function early in life (Fox, 1965; Smart and Dobbing, 1971). It also reflects the integrity of cerebellar and sensorimotor development, and of vibrissae integration (Zhang et al., 2010). Adequate reflex development depends on myelination and synapse processes, and the action of neurotransmitters (Bourre et al., 1987; Morgane et al., 1993). The anticipation of the negative geotaxis demonstrates positive evolution in labyrinth and/or vestibule function, while anticipation of cliff avoidance reflects sensorimotor function maturity (Santillán et al., 2010). Righting reflex involves both motor and visual functions (Boyle, 2001) and confirms the nervous system's maturation. Our results showed that avocado oil and pulp promoted acceleration of neonate reflex maturation. A number of experimental studies support the results of the present study for animals treated with cashew nuts (Melo et al., 2017), soybean and fish oil (Santillán et al., 2010), and goat's milk fat (Soares et al., 2013). However, our results verify that consumption of avocado pulp promotes a more pronounced acceleration in reflex; by anticipating six of the seven observed parameters. At the end of lactation, SFA and MUFA levels were lower in the brains of the animals treated with oil and pulp than in the control group; while PUFA levels were higher in the brains of the animals treated with oil (13.5%) and pulp (28%). These results suggest that high levels of PUFA may be directly related to the offspring's reflex development and the higher consumption of pulp justifies the better result observed in these groups.

The avocado used in the present research is a source of bioactive components such as phenolics, flavonoids, and carotenoids (Ameer, 2016), and the pulp has more of these compounds than the oil. These substances cross the placental barrier reaching the fetal tissue (Todaka et al., 2005), accumulating in the retina (carotenoids) and playing an important role in the development of vision and the nervous system (Hammond, 2015; Zielinska et al., 2017). Thus, neonate neuroprotection (polyphenols) (Loren et al., 2005) can induce acceleration of somatic development and reflex in the offspring (phenolics and flavonoids) (Ajarem et al., 2017). As well was observed in the present work, where both avocado oil and pulp promoted such acceleration in the development of the offspring; the results for pulp being more pronounced. An opposing result was found by Medeiros et al. (2015) D'avila et al. (2017), where the offspring of mothers supplemented with Buriti oil (rich in carotenoids), presented delayed onset for palm grasp, righting reflex and cliff avoidance reflexes.

In addition, we investigated long-lasting effects of maternal supplementation on adolescent (T45) and adult (T90) offspring, evaluating the influence of avocado consumption on animal memory. At different stages of the cycle, neurons are continuously produced in the dentate gyrus of the hippocampus, but the ontogenetic stage in which the neurogenesis occurs is crucial for memory processing. Neurons in the neonatal phase are activated through different memory processes (Tronel et al., 2015). Learning and memory processes are performed in the hippocampus dentate gyrus in cooperation with the cerebral cortex (Eichenbaum and Lipton, 2008; Coutureau and Di Scala, 2009), and PUFAs, through metabolic imprinting mechanisms affect brain functions during the development phase and promote permanent effects (van Dijk et al., 2011; Yehuda, 2012).

ARA and DHA are important constituents of membranes, especially brain tissue (Martinez, 1992; Innis, 2007) and are involved in different mechanisms that affect animal memory. DHA is involved in the expression of BNDF (brain derived neurotrophic factor), NMDA receptor (N-methyl-D-aspartate) synthesis, induction of LTP (long-term potential), and liberating glutamate in glutamatergic functions. Deficiency of ω-3 PUFA alters the fatty acid composition of the fetal brain with repercussions in the adult phase, increases fetal inflammatory processes, and induces deficits in development and memory (Labrousse et al., 2018). ARA is involved in the regulation of the cholinergic neurotransmission system and in the GABA/Glu regulatory system decreasing oxidative damage, and cellular apoptosis (Li et al., 2015). These two PUFAs were incorporated into the offsprings' brains through maternal supplementation with avocado, and ARA presented higher levels in the brains of the AO group in adolescence and the AP group as adults, while DHA presented higher levels in the AP and AO groups in adolescence and only in AP animals in adulthood.

In the present study we used the Open Field Habituation test and the Object Recognition Test (ORT) for evaluation of non-associative learning of the adolescent and adult offspring. In the Open Field Habituation test, repeated exposure to the same environment tends to cause a decrease in locomotion, recognized as a form of non-associative learning (Rachetti et al., 2012). Our results showed that in the adolescent and adult offspring, maternal supplementation with avocado oil and pulp reduced locomotion in the second exposure. The same effect has also observed in the animals fed a diet containing cashew nuts (Melo et al., 2017) and fish oil (Rachetti et al., 2012). Increased habituation, yet with memory impairment has occurred with peanut oil, containing little LA (Frances et al., 1996), but with an excess in saturated fat (Page et al., 2014).

The Object Recognition Test (ORT) involves an acquisition phase, where the rodent explores a chamber containing two similar objects and a recall phase, which occurs after a time interval in which one object is replaced by a new one. From the time interval used between the exposures, and from the ratio of time spent on the exploration of the new vs. the familiar object, and from the greater interaction with the new object, we may observe facilitation of short and long term memory (Cordner and Tamashiro, 2015); recognition of place, that involves the hippocampus (Barker and Warburton, 2011) and preference for the new object, which involves the prefrontal cortex (Mumby and Pinel, 1994; Bussey et al., 2000). Our results demonstrated that maternal supplementation with avocado oil and pulp facilitated acquisition of recognition memory in the adolescent and adult offspring, evidenced by a higher exploration rate (of the new object), both short and long term. Melo et al. (2017) has demonstrated that a maternal diet containing cashew nuts yields good short-term memory performance in the offspring. The offspring of mothers supplemented with fish oil has been shown to present good long-term memory performance (Rachetti et al., 2012). Other studies have demonstrated improvements in cognitive performance in offspring in relation to maternal consumption of olive (Pase et al., 2015) and linseed oils (Fernandes et al., 2011). However, maternal consumption of high ω-6/ω-3 ratio (Lépinay et al., 2015); saturated fats (Frances et al., 1996; Souza et al., 2012; Arcego et al., 2017), hydrogenated vegetable fat (Pase et al., 2017) and interesterified fat (D'avila et al., 2017) caused damage to the animals' memory. The results obtained in our study demonstrate that increased ARA and DHA levels in the brains of the offspring of the supplemented groups interfered directly in memory development. Yet both DHA and ARA are responsible for maintaining optimal growth and functional behavior of the offspring (Harauma et al., 2017). DHA, in particular, is capable of protecting the hippocampus against oxidative stress and apoptosis; preventing memory deficits (Gao et al., 2016).

The positive effects of maternal supplementation with avocado oil and pulp on the memory of from adolescents to adult offspring can also be explained by its antioxidant potential. Experimentally, the antioxidant action of this fruit has been proven in diabetic rats supplemented with its oil (Ortiz-Avila et al., 2015). Studies have shown that the effect of maternal consumption of flavonoids on offspring memory is associated with decreased oxidative brain damage; due to reductions in lipid peroxidation levels, and generation of reactive species, and an increase in the antioxidant defense system as well as BDNF in the adult rat pre-frontal cortex (Bussey et al., 2000), and modulation of hippocampal signaling (Corona et al., 2013).

In the present study, the fatty acid profile in the brains of the offspring of mothers supplemented with avocado oil and pulp during gestation and lactation was measured at different stages of the life cycle. The animals of the two experimental groups, oil and pulp showed better somatic maturation, an anticipation of reflexes and improvement in memory. These findings demonstrate the benefits that maternal supplementation with a source of monounsaturated fatty acids and antioxidant compounds can bring to the development of the brain, persisting into adulthood.

As a limitation of this study, the groups treated with avocado were not compared with animals treated with lipid source deficient in essential fatty acids. On the other hand, we objected with the present study to define whether maternal supplementation with pulp and avocado oil could have a distinct effect on neurodevelopment of the offspring.

## Conclusion

Maternal supplementation with avocado oil and pulp influences the development of the nervous system of the offspring in the short and long term, accelerating somatic development and reflex maturation while improving memory in the adolescent and adult phases.

## Author Contributions

This research was carried out by all authors. JS, MP, and MM designed the theme of the study. MM, RM, ES, DP, and MC performed the experimental methods designed. SS and CD performed fatty acid analysis and VV carried out analysis of the antioxidant components. JS, MO, FM, and MM analyzed the data. JS and MM interpreted the results and wrote the article.

### Conflict of Interest Statement

The authors declare that the research was conducted in the absence of any commercial or financial relationships that could be construed as a potential conflict of interest.
